# The risk of AIDS-defining events is decreasing over time in the German HIV-1 Seroconverter Cohort

**DOI:** 10.1186/1471-2334-12-94

**Published:** 2012-04-19

**Authors:** Mathias Altmann, Matthias an der Heiden, Ramona Scheufele, Katrin Hartmann, Claudia Houareau, Barbara Bartmeyer, Osamah Hamouda

**Affiliations:** 1 Dept. Infectious Diseases Epidemiology, Robert Koch Institute, Division 34 HIV/AIDS, STI and Blood-borne Infections, DGZ-Ring 1, Berlin, D-13086, Germany

**Keywords:** HIV progression, AIDS-defining event, Seroconverter, HAART effectiveness

## Abstract

**Background:**

With ageing of the HIV-infected population, long-term exposure to treatment, varying adherence, emerging resistance and complications to therapies, effectiveness of Highly Active Antiretroviral Therapy (HAART) needs to be monitored continuously at the population level. The German HIV-1 Seroconverter Cohort is a multi-centre, open, long-term observational cohort including patients with a known or reliably estimated date of HIV-infection i.e. last negative and first positive HIV antibody test within a maximum three-year interval or laboratory evidence of seroconversion. Our study aims to investigate survival improvements and changes in AIDS risk over calendar periods in the German HIV-1 Seroconverter Cohort.

**Methods:**

Retrospective (for the pre-1997 period) and prospective (since 1997) data from the German HIV-1 Seroconverter Cohort were used. Time from seroconversion to first AIDS-defining event over calendar periods was analysed by using Cox models adjusting for age at seroconversion, sex, transmission groups and short HIV test interval. Kaplan-Meier methods were used to determine expected survival (remaining AIDS-free) by calendar period.

**Results:**

2162 seroconverters with 8976 person-years of observation were included in our analysis (up to 31.12.2010). A total of 196 first AIDSdefining events were reported. Two periods i.e. 19972000 and 2007-2010 were statistically associated with a reduction in the risk of AIDS, accounting for an overall reduction of 80%. Compared to1997-2000, hazard ratios were 2.6 (95%CI, 1.6-4.8; p=0.000) in pre-1997 and 0.5 (95%CI, 0.3-0.8; p=0.007) in 20072010. Independent risk factor for AIDS progression was age at seroconversion (HR, 1.3 per 10year-increase; p=0.001).

**Conclusion:**

HAART effectiveness has improved in the German HIV-1-Seroconverter Cohort. The risk to develop AIDS decreased significantly in 19972000 and in 20072010. However, elderly may require particular monitoring in view of their faster progression to AIDS.

## Background

Long-term observational studies with reliable HIV-1 seroconversion dates are a good tool to assess the effectiveness of highly active antiretroviral therapy (HAART) at the population level [1]. They use calendar period as a proxy for actual HAART use to circumvent confounding by indication [2]. Just after the introduction of HAART in 1996, such studies showed a protective effect of HAART on time to acquired immunodeficiency syndrome (AIDS) [3-5]. Thereafter, continuous decrease on time to AIDS has been reported, coincident with the widespread uptake of HAART [6-8]. However, with ageing of the HIV-infected population, long-term exposure to treatment, varying adherence, emerging resistance and complications to therapies, there is a need to monitor regularly the effectiveness of HAART at the population level. Furthermore, the introduction of new drugs may have contributed to slowing down the disease progression in more recent times.

In Germany, HIV surveillance is regulated by the national Protection against Infection Act since 2001 [9]. Newly diagnosed HIV infections are reported to the Robert Koch-Institute (RKI), the national institute for diseases surveillance and control. However, in this surveillance system, the date of HIV infection is often unknown. To supplement the mandatory reporting system, the RKI set up in 1997 the HIV-1 Seroconverter study, which allows estimating the time of infection, leading to a reliable determination of AIDS incidence, risk factors for AIDS and time to therapy initiation.

This paper aims to present the results of the German HIV-1 Seroconverter study, covering more than 13years of surveillance, in order to monitor at the population level the progression to AIDS and associated risk factors over calendar periods.

## Methods

### Study design

The German HIV-1 Seroconverter study started in 1997 as a national multicenter observational cohort study including HIV-infected persons for whom the date of seroconversion is known or could reliably be estimated. Study participants are recruited by more than 22 outpatient clinical centres, 40 medical practices specialised on HIV/AIDS and seven local health authorities all over Germany, mainly in German metropoles. All HIV infected patients aged 18years or older (at the time of study enrolment) are eligible to be included in the cohort if their date of seroconversion can be reliably estimated, either as acute or as documented seroconverters, which has been described in detail elsewhere [10]. Briefly, acute seroconverters included individuals with laboratory evidence of seroconversion and those with an interval of maximum three months between the last negative and the first positive HIV-antibody test (mid-point between the dates is used to estimate the time of infection). Documented seroconverters included individuals with a last negative and a first positive HIV-antibody test within a maximum three-year interval (mid-point between the dates is used to estimate the time of infection).

### Data collection

On the basis of a baseline standardized paper-based questionnaire, demographic (including sex, age, and HIV exposure category), clinical (including CDC-status and current therapy regimes) and laboratory data (including viral load, CD4+ and CD8+ counts) are collected by physicians. Yearly follow-up questionnaires include CDC-status and current therapy regimes. Questionnaires are sent to the RKI where they are reviewed and integrated in the HIV database, and checked for completeness and plausibility. If data inconsistencies cannot be resolved, the institutions are queried.

### Statistical analyses

We analysed the time from seroconversion to AIDS via Cox proportional hazard model, a method that allows for late entry and inclusion of retrospectively ascertained seroconversion [11]. Since the objective of these analyses was to measure the effectiveness of HAART at the population level, calendar year at risk was divided into five calendar periods, according to the availability of new anti-retroviral drugs in Germany: 1) the era before HAART (pre-1997), 2) limited use of HAART and use of two Nucleoside Reverse Transcriptase Inhibitors (NRTI) with one Protease-Inhibitor (PI) or one Non-Nucleoside Reverse Transcriptase Inhibitors (NNRTI) (19972000), 3) introduction of Lopinavir/Ritonavir as boosted PI in one co formulation (20012004), 4) introduction of secondary PI generation (20052006), and 5) the introduction of Chemokine Coreceptor 5 (CCR5) antagonists and integrase single strand transfer inhibitors (20072010). Calendar period was included as a time-dependent covariate so that each individual contributed to the analyses with all time periods they had been at risk. Time was always measured from date of seroconversion so that comparisons across calendar periods were only based on seroconverters who had been infected for the same length of time. The second calendar period (19972000) was used as reference period in the Cox proportional hazard model.

Individuals were censored either by the day of the last medical visit or by the date of the first AIDS-defining event, defined in accordance with clinical criteria from the 1993 Centers for Disease Control and Prevention (CDC) case definition [12]. Individuals with pre-AIDS mortality were censored as AIDS free at the moment of death. Lost to follow up was defined as the number of last medical visits in each calendar period. Due to a delay of reporting, last medical visits in 2010 were not considered as lost to follow up but as unknown status.

Adjustment was done for sex, HIV exposure category, age at seroconversion and short HIV test interval (already defined as acute seroconverters). HIV exposure categories included, in the following order of priority: injecting drug users (IDU), men who have sex with men (MSM), heterosexuals, other (including blood transfusion and occupational acquisition), and unknown. Among heterosexuals, a new group including people originated from high prevalence countries (HPC) was created. To investigate whether the effects of these determinants changed over time, we included interaction terms between each determinant and the calendar periods at risk. Interaction was tested using likelihood ratio tests. Kaplan-Meier methods were used to determine the expected survival probability (remaining AIDS-free) in each calendar period and Log-rank tests for equality of survivor functions. All p-values are two-sided, and a p-value of 0.05 or less was considered significant. All data were analysed using STATA 11.0 (StataCorp LP, College Station, TX, USA).

### Ethics

The Seroconverter study was approved by the local ethics committee of the Charit (University hospital) in Berlin and reconfirmed in 2010. Informed consent was obtained for each participant of the study.

## Results

### Study population

By the end of 2010, a total of 2162 eligible patients were enrolled in the study and included in the analysis. Of these, 214 patients were retrospectively included, namely having a date of seroconversion before 1997. Both the number of included patients and follow-up increased largely after 2001, when the study was better established. The overall median age at seroconversion was 33years (IQR: 2739) without major changes over calendar periods (Table 1). The main exposure category was MSM, representing 85.5% (1838/2162) of the study population and resulting in a low proportion of women in the cohort (137/2162; 6.3%). The MSM proportion increased as a proportion of enrolled seroconverters in later calendar periods (65.0% in pre-1997 to 87.6% in 20072010). The second main HIV exposure category was heterosexual contacts, representing 9.0% (195/2162) of the patients. IDU represented only 2.6% (55/2162) of the seroconverters and were decreasingly enrolled over time. 1.5% (32/2,162) of the patients originated from high prevalence countries. More than 40% of the study population had a short HIV test interval and this proportion increased over time. For those including in the documented seroconverters (HIV test interval bigger than 3months), the HIV test interval was between 3 and 12months.

**Table 1:** Description of the German HIV-1 Seroconverter Cohort by the end of 2010

	**Calendar year of seroconversion**					
	**Pre-1997**	**1997-2000**	**2001-2004**	**2005-2006**	**2007-2010**	**Overall**
Sex, female, n (%)	32 (15)	13 (7.0)	32 (5.6)	29 (6.2)	31 (4.2)	137 (6.3)
Age at seroconversion, (year) [median (IQR)]	30 (2638)	32 (2736)	33 (2738)	33 (2840)	33 (2740)	33 (2739)
HIV exposure category [n (%)]						
men who have sex with men	139 (65.0)	152 (82.6)	500 (88.2)	406 (87.7)	641 (87.6)	1838 (85.0)
Injecting drug users	32 (15.0)	5 (2.7)	10 (1.8)	5 (1.1)	3 (0.4)	55 (2.6)
heterosexuals	34 (15.9)	16 (8.7)	40 (7.1)	41 (8.8)	64 (8.8)	195 (9.0)
people from high endemic country	6 (2.8)	6 (3.2)	9 (1.6)	5 (1.1)	7 (1.0)	33 (1.5)
others*	0 (0)	2 (1.1)	2 (0.4)	1 (0.2)	4 (0.6)	9 (0.4)
unknown	3 (1.4)	3 (1.6)	6 (1.1)	7 (1.5)	13 (1.8)	32 (1.5)
Short HIV test interval , n (%)	33 (15.4)	53 (28.7)	206 (36.3)	208 (44.7)	401 (54.9)	901 (41.7)
Seroconverters, n (Follow up)	214 (520)	185 (837)	567 (1201)	465 (1259)	731 (5090)	2162 (8906)
AIDS events, n, (rate)	36 (6.9)	22 (2.6)	23 (1.9)	47 (3.7)	68 (1.3)	196 (2.2)
Pre-AIDS mortality, n (rate)	NA	5 (0.6)	4 (0.3)	4 (0.3)	14 (0.3)	27 (0.3)
Lost to follow-up, n (rate)	2 (0.4)	85 (10.2)	169 (14.1)	126 (10.0)	303 (10.5)	685 (10.2)

* Occupational and blood transfusion.

† Defined as 3months between last negative and first positive HIV test, or with laboratory evidence of seroconversion.

‡ Follow up in person-year.

± Rate calculation: number of events/100 person-years.

IQR: Interquartile range; NA: Not available.

### AIDS-defining events, incidence rate and survival

Over the 8906 person-years (PY) of follow up (median=2.8years; range 1day 23.9years), 196 first AIDS-defining events were reported, leading to an overall AIDS incidence rate of 2.2 per 100 PY (95% CI, 1.9-2.5). The AIDS incidence rate per 100 PY decreased significantly from 6.9 in pre-1997 (95% CI, 4.9-9.6) to 2.6 (95% CI, 1.7-4.0) in 19972000, 1.9 (95% CI, 1.3-2.9) in 20012004, 3.7 (95% CI, 2.8-5.0) in 20052006 and 1.3 (95% CI, 1.1-1.7) in 20072010 (Table 1). Among the 196 reported AIDS-defining events, opportunistic infections (139/196, 71%) were the most prevalent, followed by Kaposi's sarcoma (21/196, 11%), lymphoma (15/196, 8%), HIV encephalitis (11/196, 5%) and cachexia (10/196, 5%).

**Figure 1:**
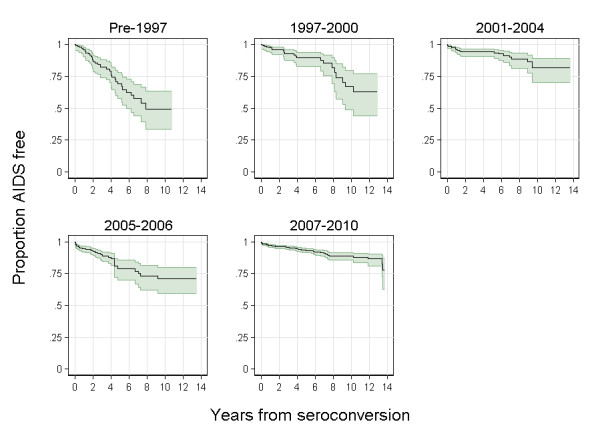
Proportion of AIDS free patients (with 95% Confident Interval) after HIV seroconversion over time, by calendar period, Germany.

The proportion of patients developing AIDS within 2 and 10years decreased significantly (p=0.0001) from 14% and 50% in pre-1997 to 4% and 33% in 19972000, respectively (Figure 1). The two following periods did not show a decrease in the proportions of patients developing AIDS within 2 and 10years in comparison to 19972000. In the last period (20072010), these proportions further decreased significantly (p=0.0129) to 4% and 11%, respectively.

### Pre-AIDS mortality and lost to follow-up

Death without AIDS, which accounted for 27 of the cases, had a relatively stable rate over calendar periods after 1997 (as this information was not retrospectively collected for the pre-1997 period), from 0.6 per 100 PY in 19972000 to 0.3 per 100 PY in the following periods. The lost to follow up rate was also relatively stable over the calendar periods, except for the pre-1997 period where it was lower (0.4 per 100 PY) (Table 1).

### Calendar periods and prognostic factors

In the multivariable model adjusted for sex, age at seroconversion, HIV exposure category and short HIV test interval, two periods i.e. 19972000 and 20072010 were statistically associated with a reduction in the risk of AIDS, accounting for an overall reduction of 80%. Compared to 1997-2000, hazard ratios were 2.6 (95%CI, 1.6-4.8; p=0.000) in pre-1997 and 0.5 (95%CI, 0.3-0.8; p=0.007) in 20072010 (Table 2). Both the 20012004 and 20052006 periods did not show significant differences in the risk of AIDS compared to the 19972000 period, but only a slight non-significant increase in 20052006.

**Table 2:** Adjusted Hazard Ratios of progression to first AIDS-defining event in the German HIV-1 Seroconverter Cohort, 2010

	**HR† **	**95%CI**		**p-value**
Calendar Period				
Pre-1997	2.6	1.6	4.8	0.000
1997-2000	1.0	1.0	1.0	-
2001-2004	0.7	0.4	1.2	0.191
2005-2006	1.3	0.8	2.2	0.316
2007-2010	0.5	0.3	0.8	0.007
Sex, women	1.2	0.6	2.3	0.544
Age (per 10years increase in age)	1.3	1.1	1.5	0.001
HIV exposure category				
men who have sex with men	1.0	1.0	1.0	-
Injecting drug users	1.4	0.8	2.7	0.229
heterosexuals	0.8	0.4	1.5	0.479
people from high endemic country	2.3	1.0	5.3	0.053
others*	1.1	0.2	8.0	0.910
unknown	1.6	0.6	4.4	0.340
Short HIV test interval ‡	1.3	1.0	1.8	0.056

* Occupational and blood transfusion.

† adjusted for calendar period, sex, age at seroconversion, HIV exposure category, short HIV test interval.

‡ defined as 3months between last negative and first positive HIV test, or with laboratory evidence of seroconversion.

HR: Hazard Ratio; CI, confident interval.

Older age at seroconversion was associated with an increased risk of AIDS (HR, 1.3 per 10year-increase; p=0.001). Compared to the group of MSM, the risks among people originating from HPC and IDU were not significantly increased, with HR of 2.3 (95%CI 1.0-5.3; p=0.053) and 1.4 (95%CI, 0.8-2.8; p=0.229), respectively. Heterosexual transmission had a similar risk as compared with homosexual male transmission (HR, 0.8; 95% CI 0.4-1.5; p=0.479).

To investigate whether the prognostic effects of these covariates had changed over time, we included interactions between calendar period and each of the covariates in the Cox model. To reduce model instability, we collapsed the calendar periods to pre-1997, 19972006 and 20072010, and excluded 9 patients with occupational exposure and 36 with unknown exposure. There was no evidence of a change over time in the effect of age (p=0.1287) although we noticed a decrease in the HR from 1.6 to 1.0 over the calendar periods (Table 3). There were no significant changes in the effect of sex and short HIV test interval. Although HIV exposure categories did not change over time (p=0.5575), the risk of AIDS decreased less among IDU than for the other HIV exposure categories over the calendar periods.

**Table 3:** Interactions between prognostic factors and calendar periods in the German HIV-1 Seroconverter Cohort, 2010

				**Calendar periods**			**P-value for interaction**
		**pre-1997**		**1997-2007**		**2007-2010**	
	**HR† **	**95% CI**	**HR† **	**95% CI**	**HR† **	**95% CI**	
Sex, female	1.0	0.2-4.6	1.7	0.8 3.7	0.7	0.1 4.5	0.6509
Age (per 10years increase in age)	1.6	1.1-2.2	1.4	1.2 - 1.8	1.0	0.8 - 1.4	0.1287
HIV exposure category							
men who have sex with men	1		1		1		
Injecting drug users	0.8	0.3 - 2.6	1.9	0.9 4.1	1.4	0.2 11.1	0.5575
heterosexuals	0.2	0.04 - 1.4	1.1	0.5 - 2.4	0.5	0.1 - 2.1	
people from high endemic country	2.7	0.6 12.7	2.1	0.7 - 7.0	1.6	0.2 13.8	
Short HIV test interval*	1.4	1.0 1.9	1.1	0.7 - 1.8	1.7	1.0 2.8	0.3956

*defined as 3months between last negative and first positive HIV test, or with laboratory evidence of seroconversion.

† To ease the interpretation we show all hazard ratios in comparison to the MSM risk of the respective calendar period.

HR: Hazard Ratio; CI, confident interval.

### Therapy/time to therapy

**Figure 2:**
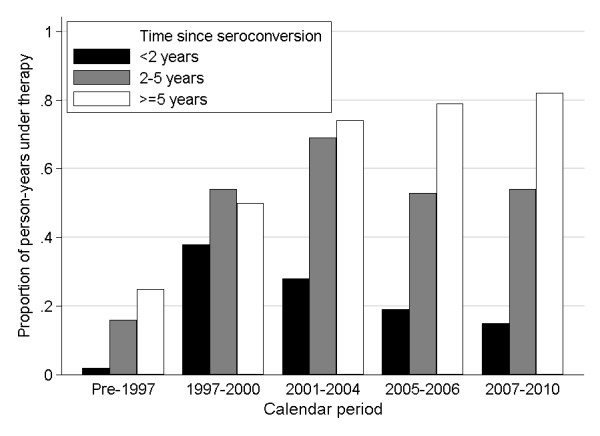
Proportion of person-year (PY) under therapy over the total PY follow up from seroconversion, stratified by time from seroconversion and calendar period, Germany.

While mono and dual NRTI therapy were mostly used before 1997, main combinations of HAART included 2 NRTI/PI till 2001 and a mixed of 2 NRTI/PI and 2 NRTI/NNRTI after this date. While the proportion of PY spent on antiretroviral therapy was 51% for the whole cohort, this proportion changes over the calendar periods, from 13% in pre-1997 to 48%, 59%, 45% and 54% in 19972000, 20012004, 20052006 and 20072010, respectively. The median time from seroconversion to therapy initiation was 1.3years [interquartile range (IQR), 0.5-2.8years]. The temporal trend of this median showed first a decrease from 1.6 in pre-1997 to 0.8 and 0.4years in 19972000 and 20012004 respectively, followed by an increase to 1.4 and 1.9years in 20052006 and 20072010 respectively. More precisely, this trend was confirmed when analysing proportions of person-years spent on treatment over total follow-up time, stratified by calendar period and categorised by the proportion initiating treatment within 2years, between 2 and 5years, and more than 5years (Figure 2). Whilst the proportion of person-years on treatment within 2years after seroconversion decreased since 1997, the proportion of patient-years on treatment 5years and more after seroconversion increased from 25% in pre-1997 to 82% in 20072010. The proportion initiating treatment between 2 and 5years after seroconversion was more constant and varied between 53 and 69% since 1997.

## Discussion

By using the data from the German HIV-1 Seroconverter Cohort, we estimated the risk of AIDS in calendar periods with different HAART regimens as well as during mono and dual antiretroviral therapy (pre-1997). We found an overall reduction of 80% in the risk of AIDS over the calendar periods, which was not linear but included two main drops, one after 1997 and one after 2007. This is the first study to our knowledge showing a reduction in AIDS risk after 2007. However, these results might be influenced by the fact that the study population includes a large proportion of MSM who are more likely to be closely monitored by HIV specialists and may therefore not be representative of the entire German population infected by HIV. A similar cohort study in Spain reported significant reduction in the risk of AIDS over calendar periods in comparison with the pre-HAART era, but there were no differences between the calendar periods in the post-HAART era [8]. However, the last observed calendar period was 20002003 and there were more IDU in the composition of this cohort than in our study. In another study combining 22 cohorts of people living with HIV-1 from Europe, Australia and Canada, the authors showed an even bigger reduction in the risk of AIDS, with a relative risk of 0.46 and 0.13 after 1997 and after 2001, respectively [6].

Our results showed an overall increase in HAART uptake, which likely parallels the decrease in AIDS risk. These trends have also been seen in the UK, where the proportion of patients being treated by antiretroviral therapy increased from less than 2% in pre-1996 to 58% in 20042006 [7]. The decrease in HAART uptake in 20052006 in our study, associated with an increase in AIDS risk and AIDS incidence in the same period, supports the hypothesis of this association between HAART uptake and AIDS risk. Increasing HAART uptake benefits could have been balanced by an increasing prevalence of Transmitted Drug Resistance (TDR) over the calendar periods. However, one previous study in the same cohort of seroconverters indicated a stable prevalence of TDR over the time [13]. Other factors related to the improvements in drug quality, including safety and galenic formulation easing intake, together with increases in HIV-knowledge among health professionals have probably also contributed to the decrease in AIDS risk.

We observed a trend towards initiating treatment later following seroconversion in later calendar periods. This could be explained by the different recommendations in Germany since 1997. Following the Vancouver Conference [14] and the increasing uptake of HAART, the main attitude in Germany was to hit hard and early [15]. In 2002, following the large proportion of people with antiretroviral therapy side effects/toxicity, the German AIDS Society recommended to start therapy below a threshold of 200 CD4+ cells per L [16]. This recommendation might partially explain the slight decrease in HAART uptake and consequently, the slight increase in AIDS risk for the period 20052006 in our study. In 2008, recommendations were revised to initiate the antiretroviral therapy below a threshold of 350 CD4+ cells per L [17]. Surprisingly, this last recommendation was not associated with an increase, neither in the median time to treatment initiation nor in the proportion of people being treated less than 2years and between 2 and 5years after seroconversion. However, this last period was associated with a decrease in AIDS risk, indicating that factors other than time to treatment initiation may play a more important role in disease progression.

The main determinant that affects disease progression in our cohort was age at seroconversion. This factor, together with duration of infection, was already known to be a crucial determinant of HIV disease progression in developed countries before the introduction of HAART [5]. However, age disparities seem to diminish over the calendar periods in our cohort, as already shown in another study including 22 cohorts [6].

Our results did not show an effect of sex on AIDS risk. This result contrasts with recent studies that reported a slower disease progression among women than men [8,18]. However, the number of women in our study population was too small to detect any potential differences.

Comparing disease progression rates between transmission risk groups in our study was difficult, considering the large proportion of MSM compared to the other transmission groups. However, the trend over the calendar periods showed that IDU had a smaller reduction in AIDS risk as compared with the others risk groups. This result is supported by Porter et al., reporting a smaller reduction in AIDS risk in the post- HAART era among IDU [6]. However, other causes of mortality, such as hepatitis C, could confound this result. People from high HIV prevalence countries had at higher, although non-significant risk for progression to AIDS. Furthermore, the risk seems to decrease with the calendar periods. This result should reflect a heterogeneous population and be interpreted with caution because of the small number of people in this group. Nevertheless, access to health services, health insurance status, poor adherence to treatment [19] and other co-morbidities could have played a role and may require special attention for this group.

The presence of seroconversion illness, typically characterized by flu-like symptoms, has been reported to be associated with a more rapid disease progression [20,21]. In order to avoid recall bias, short HIV test interval has been reported to be a good proxy for the presence of seroconversion illness [21]. In our study, short HIV test interval factor showed a slight effect, which appears to increase over time. However, this result depends on the quality of the tests used and on the time interval chosen to define a short test interval.

This studys principal strength is its good quality data on date of seroconversion, permitting reliable approximation of AIDS incidence and risk. This study also has the advantage of providing data for more than 13years of prospective follow-up from different settings, representing roughly 10% of all new HIV diagnoses reported to the national HIV surveillance since 2004. However, some limitations have to be raised. First, the data for the pre-1997 period are retrospective and may not be as reliable as the data from the prospective period. Patients in this period were included regardless of their AIDS outcome; a survivorship bias might have been introduced as patients have to sign an informed consent at study entry. Regarding the pre-AIDS mortality, this bias might have been limited as the pre-AIDS mortality rates were relatively stable and at a very low level during the prospective periods. Regarding the lost to follow up, rates were relatively stable over the time periods but were at a high level. If lost to follow up subjects have a more rapid progression, the pre-1997 period in our study might underestimate the risk of AIDS in comparison to the other periods but our results remain conservative to this regard. Alternatively, we might have overestimate the first decrease in the AIDS risk if lost to follow up subjects have a slower disease progression. Considering only the periods after 1997, a bias might have been introduced if lost of follow up subjects differ between the periods. Further analyses (e.g. competing risk analysis) should be performed to better answer that issue. Another issue is that women are largely underrepresented in this study (6.2%) in comparison with the national reporting system, in which women accounted for as many as 20% of all new diagnoses between 1997 and 2010. This proportion does not allow us to analyze the effects of sex and HIV transmission categories with enough power. Finally, we assumed continuous HAART intake after treatment initiation. This limitation might be important for the interpretation of the proportion of people being treated by calendar period. However, this does not change the interpretation of the effectiveness over time. The treatment was considered to be HAART (i.e., three or more drugs that are from two or more classes, or that contain abacavir) in every calendar period from 1997 onwards, even if this might not be the case for one or two percent.

## Conclusions

HAART effectiveness has improved in the German HIV-1-Seroconverter Cohort. After a main reduction in the risk of AIDS in 19972000, a second significant decrease occurred in 20072010. However, elderly may require particular monitoring in view of their faster progression to AIDS.

## Abbreviations

AIDS: Acquired immunodeficiency syndrome; CDC: Centres for Disease Control and Prevention; HAART: Highly Active Anti Retroviral Therapy; HPC: People from high HIV prevalence countries; HR: Hazard ratio; IDU: Injecting drug users; IQR: Interquartile Range; MSM: Men who have sex with men; NRTI: Nucleoside reverse transcriptase inhibitor; NNRT: Non- nucleoside reverse transcriptase inhibitor; PI: Protease inhibitor.

## Competing interests

The authors declare that they have no competing interests.

## Author contributions

All authors were involved in asking the research question presented in this manuscript. MA was responsible for statistical analyses together with MadH. MA wrote the first draft of the paper. All authors reviewed the final manuscript.

## Source of funding

The German HIV-1 Seroconverter Cohort is funded by the German Ministry of Health.

## Pre-publication history

The pre-publication history for this paper can be accessed here:

http://www.biomedcentral.com/1471-2334/12/94/prepub
